# Cannabidiol-Loaded Nanoparticles Based on Crosslinked Starch: Anti-Inflammatory Activity and Improved Nose-to-Brain Delivery

**DOI:** 10.3390/pharmaceutics15071803

**Published:** 2023-06-23

**Authors:** Ilya Eydelman, Na’ama Zehavi, Valeria Feinshtein, Dinesh Kumar, Shimon Ben-Shabat, Amnon C. Sintov

**Affiliations:** 1 Department of Clinical Biochemistry and Pharmacology, Ben-Gurion University of the Negev, Be’er Sheva 8410501, Israel; eydelman@bgu.ac.il (I.E.); shteiman@bgu.ac.il (V.F.); dineshbarbola@cuh.ac.in (D.K.); 2 Laboratory for Biopharmaceutics, E.D. Bergmann Campus, Ben-Gurion University of the Negev, Be’er Sheva 8410501, Israel; naamaz1974@gmail.com; 3 Department of Pharmaceutical Sciences, Central University of Haryana, Mahendragarh 123031, Haryana, India; 4 Department of Biomedical Engineering, Ben Gurion University of the Negev, Be’er Sheva 8410501, Israel

**Keywords:** cannabidiol nanoparticles, intranasal delivery, inflammation, microglia cells, nitric oxide, interleukin-6

## Abstract

Cannabidiol (CBD) has previously been shown to inhibit inflammatory cytokine production in both in vitro and in vivo studies of neurodegenerative diseases. To date, the CBD treatment of these diseases by quantitative targeting directly to the brain is one of the greatest challenges. In this paper, we present a new particulate system capable of delivering CBD into the brain via the intranasal route. Intranasal administration of CBD-loaded starch nanoparticles resulted in higher levels of cannabidiol in the brain compared to an identically administered cannabidiol solution. The production and the characterization of starch-based nanoparticles was reported, as well as the evaluation of their penetration and anti-inflammatory activity in cells. Cannabidiol-loaded starch nanoparticles were prepared by crosslinking with divanillin, using the nanoprecipitation method. Evaluation of the anti-inflammatory activity in vitro was performed using the BV2 microglia cell line. The starch nanoparticles appeared under electron microscopy in clusters sized approximately 200 nm in diameter. In cultures of lipopolysaccharide-induced inflamed BV2 cells, the cannabidiol-loaded starch nanoparticles demonstrated low toxicity while effectively reducing nitric oxide production and IL-6 levels. The anti-inflammatory effect was comparable to that of a glucocorticoid. Starch-based nanoparticle formulations combined with intranasal administration may provide a suitable platform for efficacious cannabidiol delivery and activity in the central nervous system.

## 1. Introduction

Neurological dysfunctions in the central nervous system (CNS) disrupt memory, speech, and sensorimotor and autonomic performance, with different degrees of severity. Although neurological disorders can be caused by degeneration, trauma, infection, neoplasm, or autoimmune condition [1], neuroinflammation is common in most disorders [2]. Therefore, inhibition of CNS inflammation is optional for many of these conditions. Indeed, this strategy produced a neuroprotective effect in various studies [3,4]. Neuroinflammation is a term that describes several cellular and molecular pathologies that result from the activation of resident CNS cells, and infiltration of CNS by leukocytes or by inflammatory mediators within the CNS. The principal CNS cell types involved in the inflammatory processes are microglia and astrocytes. Lymphocytes, neutrophils, and monocytes from peripheral blood can infiltrate CNS and escalate neuroinflammation. Both resident and infiltrating cells produce inflammatory mediators, particularly cytokines, which are crucial in the inflammation processes [5]. Transient or moderate inflammation can be beneficial, but prolonged or excessive microglia activation may result in pathologic inflammation and contribute to neurodegenerative and neoplastic diseases [6]. Although drug-targeting various inflamed tissues in the CNS has been demonstrated as a treatment option, the delivery of therapeutic substances to the brain is not an easy task. Due to low drug permeability and cellular uptake, new methods have been sought to effectively deliver active agents, such as cannabidiol, to the CNS tissues without causing undesired consequences. Cannabidiol (CBD) is one of the important phytocannabinoids isolated from the plant *Cannabis sativa*. It lacks psychoactive effects and has a good safety profile [7]. Cannabidiol has been reported to have pharmacological effects in several pathology models, ranging from inflammatory and neurodegenerative diseases to epilepsy, autoimmune disorders, arthritis, schizophrenia, and cancer [8]. Various in vitro and in vivo evidence supports the role of CBD in treating degenerative inflammatory diseases. It can strongly inhibit the production of inflammatory cytokines, including IL-1β, IL-6, and interferon β in LPS-stimulated murine microglial cells [9]. Despite its therapeutic potential, the successful delivery of CBD remains a significant challenge. After an oral administration and a first-pass effect, the bioavailability of CBD is between 13% and 19% [8]. Therefore, an effective delivery platform based on nanotechnology can significantly increase the CBD quantities delivered into the CNS. Intranasal administration of nanoparticulate systems is an alternative solution to the conventional delivery of CNS-targeted drugs. The nasal cavity can be divided into respiratory and olfactory regions. In the respiratory region, drugs can enter systemic circulation or be directly transported to the brain tissue through the trigeminal nerve pathway. In the olfactory region, drugs can be transported or diffused directly to the brain through the olfactory mucosa pathway, considered the most important direct pathway [10]. The use of various drugs delivered through nanoparticulate platforms via the intranasal route has been described as a potential strategy for overcoming the challenges of drug delivery to the brain [10,11,12].

In the present study, we have demonstrated that nanoparticles based on crosslinked starch (starch nanoparticles, SNPs) are capable of delivering relatively high quantities of CBD into the brain via the intranasal route compared to an intranasal non-particulate CBD. We manufactured CBD-loaded SNPs by a nanoprecipitation technique [13,14,15], during which crosslinking of starch by divanillin (DV) took place [15]. After characterization of these SNPs, we evaluated their intracellular uptake, cell viability, and the anti-inflammatory effect in an LPS-induced inflammation model of microglia cells.

## 2. Materials and Methods

### 2.1. Materials

Maize (corn) starch was obtained from Hopkin & Williams Ltd. (Chadwell Heath, Essex, England). Divanillin (DV) was prepared by an enzymatic reaction of vanillin (Sigma-Aldrich Israel Ltd., Rehovot, Israel) in the presence of horseradish peroxidase and hydrogen peroxide. Mannitol and Triton X-100 were obtained from Sigma-Aldrich, and urea from E. Merck (Darmstadt, Germany). Sodium hydroxide AR, hydrochloric acid 32% AR, and ethyl alcohol AR were purchased from BioLab Ltd. (Jerusalem, Israel). High-performance liquid chromatography (HPLC)-grade solvents were obtained from J.T. Baker (Mallinckrodt Baker, Inc., Phillipsburg, NJ, USA).

### 2.2. Preparation of Starch Nanoparticles

Starch slurry was prepared using corn starch (1 g), sodium hydroxide (0.8 g), urea (1 g), and double-distilled water (50 mL). All components were stirred until complete dissolution (1-2 h, at room temperature). Prior to particle preparation, starch slurry was acidified to pH 3 by a concentrated HCl solution (32%). An aliquot of the acidified starch slurry containing 20 mg of starch was then diluted with purified water, and appropriate volumes of divanillin suspension (5 mg/mL) were added. Using the “non-solvent dropping” technique, ethanolic solution of CBD (2mg or 5mg/15.4 mL) was dripped into an aqueous mixture of starch and divanillin using a syringe pump (NE-300, New Era Pump Systems, Farmingdale, NY, USA) at a rate of 0.2 mL/min (20 mL syringe with 23G needle). The mixture was constantly stirred at 700 rpm by a magnetic bar in a 40 °C heated bath. At the completion of the non-solvent dripping, the mixture was stirred for an additional hour (500 rpm) and then the remaining ethanol was evaporated by a rotary evaporator (R-205 Rotavapor, Büchi Labortechnik AG, Switzerland). Purified water was added (ad 45 mL) and the starch nanoparticles dispersion was centrifuged (250 rcf, 8 °C, 4 min). The supernatant was carefully collected (40 mL) and 20 mL of mannitol solution (0.1% *w*/*v*) was added. The SNP dispersion was then lyophilized.

### 2.3. Determination of CBD Content in SNPs

Accurately weighed (3–4 mg) lyophilized CBD-loaded SNP powder was dispersed in water/methanol (1:1), sonicated for 8 min, and centrifuged at 16,000 rcf for 12 min. The supernatant was collected (800 µL) and analyzed by HPLC. The extract was injected into a HPLC system (1260 Infinity II, Agilent Technologies Inc., Santa Clara, CA, USA) equipped with a prepacked column (ReproSil-Pur 300 ODS-3, 5 μm, 250 mm 4.6 mm, Dr. Maisch, Ammerbuch, Germany). The samples were chromatographed using a mobile phase consisting of acetonitrile/acetic acid 0.1% (80:20) at a flow rate of 1 mL/min. Calibration curves—peak areas measured at 208 nm for CBD versus drug concentration—were constructed by running standard drug solutions in methanol for each series of chromatographed samples. The limit of detection (LOD) was 0.01 µg/mL and limit of quantification (LOQ) was 0.05 µg/mL. The entrapment efficiency percentage (EE%) was calculated according to the following equation:

$$\mathrm{EE}\%=\frac{Mass\;of\;CBD\;in\;formulation}{Total\;mass\;of\;CBD\;used\;in\;formulation}\;\times 100$$

*Nanoparticle tracking analysis (NTA)*: Measurements were performed using a NanoSight NS300 instrument (Malvern Instruments Ltd., Worcestershire, UK), equipped with a 642 nm red laser module and 450 nm long-pass filter, and a camera operating at 25 frames per second, which captured a video file of the particles moving at a constant flow rate from a syringe on a syringe pump. The software for capturing and analyzing the data (NTA 2.3 and 3.4) calculated the hydrodynamic diameters of the particles by using the Stokes–Einstein equation.

### 2.4. Transmission Electron Microscopy (TEM)

TEM images were recorded with FEI Tecnai T12 G2 TWIN transmission electron microscope operating at 120 kV. Samples of SNPs dispersions were deposited on a copper 300 mesh grid, coated with Formvar and carbon (Electron Microscopy Sciences, Fort Washington, PA, USA) and dried for 5 min. Samples were then stained with 1% uranyl acetate solution. Images were taken using TemCam—F214 (Gauting, Germany).

### 2.5. Cell Culture Assays

BV-2 cells were maintained in RPMI 1640 medium supplemented with 10% fetal bovine serum (FBS) and antibiotics (penicillin 100 U/mL, streptomycin 100 µg/mL) at 37 °C in a humidified incubator under 5% CO_2_.

### 2.6. Cell Viability Assay

BV2 cells were seeded in a 96-well plate at 1 × 10^4^ cells/well for normal growing conditions, and at 2 × 10^4^ cells/well for LPS-induced inflammation conditions. After 24 h, the cells were treated either with unloaded or with CBD-loaded SNPs. SNPs were dispersed in RPMI. After an additional 24 h, the medium was removed and, CellTiter 96^®^ Aqueous One Solution reagent [3-(4,5-dimethylthiazol-2-yl)-5-(3-carboxymethoxyphenyl)-2-(4-sulfophenyl)-2H-tetrazolium, inner salt; MTS] and an electron coupling reagent (phenazine ethosulfate; PES)–20 µL of reagent in 100 µL of fresh medium were added to each well. After 1 h of development, the plate was scanned by a microplate reader (iMark, Bio-Rad) at 490 nm. For viability determination in NO examination (Section 2.9), cells were seeded in 24-well plates at 2 × 10^5^ cells/well; following the induction of inflammation and treatment for 22 h, the medium was removed for examination from each well and immediately replaced with 360 µL of fresh medium containing 60 µL of solution reagent as mentioned previously. After 30 min of development, a total volume of 240 µL from each well was transferred to a 96-well plate where it was equally divided among two wells (120 µL each) and scanned by a microplate reader. An average reading was used for calculation of viability.

### 2.7. Cannabidiol Release from SNPs in Culture Medium

Two aliquots of SNP powder containing 112 µg of CBD were placed in two glass beakers, each containing 50 mL of culture growth medium. The content of one container was allowed to be unstirred whereas the other was continuously stirred at 150 rpm with a magnetic bar. Both containers were kept at 37 °C during the experiment. Samples of 5 mL were taken from each container at 2 h, 6 h, and 24 h after application. The samples were ultracentrifuged at 30,000 rcf for 12 min at a temperature of 8 °C, and the supernatant from each sample was analyzed by HPLC.

### 2.8. Cellular Uptake of Nanoparticles

BV2 cells were seeded in 6-well plates at 5 × 10^5^ cells/well. After 24 h incubation, the cells were treated with CBD-loaded SNPs dispersed in RPMI and incubated for an additional 24 h. Cell viability was determined by trypan blue staining and the concentration of cells was measured by a cell counter (Countess™ 3 Automated Cell Counter, Invitrogen). The culture medium was removed, and the cells were washed twice with phosphate-buffered saline solution (PBS). Further, the cells were dissociated from the plate by a rubber scraper. The collected cells were dispersed in PBS and divided into three equal fractions which were treated separately with PBSx1, Triton-X 100 1%, and methanol 100% to evaluate CBD in the cell surface, cytosol only, and the whole cell, respectively. The fractions were shaken for 1 h at 30 rpm using a vertical shaker (Rotamix RM1, Elmi), then centrifuged at 750 rcf for 5 min, and the supernatant was collected and analyzed for CBD content by HPLC.

### 2.9. Nitric Oxide (NO) Formation

Cells were seeded in 24-well plates at 2 × 10^5^ cells/well. Following 24 h of incubation, the cells were treated with FBS-free medium for 4 h, referred as a “starvation period”. Then, this medium was removed, and inflammation was induced by 7 ng/mL lipopolysaccharide (LPS) in a growth medium containing 1% FBS, 1% HEPES buffer, and 0.1% bovine serum albumin (BSA). The effect of various treatments—which were administered concomitantly—on NO formation was studied after 22 h of treatment using Griess reagent. Duplicates of 100 µL medium were loaded on 96-well plate together with preprepared nitrite standards (NaNO_2_). The experimental medium was used as “standard zero”. Aliquots (100 µL) of Griess reagent were added to each well. After 15 min of settling in the dark, the plate was scanned by a microplate reader (iMark, Bio-Rad) at 540 nm.

### 2.10. IL-6 Determination

The experiment was conducted as described in Section 2.9. The medium was either examined immediately at the end of the experiment or was kept at −80 °C until examination. A mouse IL-6 ELISA MAX set (BioLegend, San-Diego, CA, USA) was used to determine IL-6 levels. Standards and samples were treated according to manufacturer’s protocol.

### 2.11. Animals

All animal treatments followed protocols reviewed and approved by the Institutional & Use Committee, Ben-Gurion University of the Negev, which complies with the Israeli Law of Human Care and Use of Laboratory Animals. Sprague Dawley rats (male, 200–300 g body weight, Envigo RMS, Ein Karem, Jerusalem, Israel) were used in this study. All animals were housed in polycarbonate cages and maintained on a 12/12 h light/dark cycle under controlled temperature and humidity conditions. The rats had ad libitum access to food and water.

### 2.12. Intranasal Administration (IN)

Animals were randomly divided into groups for each formulation or post-administration period. The IN-administered dose used for each animal was 100 µg CBD in nanoparticles, which accounted for approximately 0.4 mg/kg body weight. SNP powder was suspended in water immediately before nasal installation. The applied volume was 20 μL/nostril (total of 40 μL containing 100 µg CBD). As a non-particulate control, CBD was dissolved in 50:35:15 (*v*/*v*) PEG 400:saline 0.9%:ethanol as described by Paudel et al. [16]. Similar to the SNP-administered group, the applied volume was 20 μL/nostril, which corresponded to a total dose of 100 µg CBD. The animals were sedated with isoflurane vapor just before administration. After a predetermined period from administration (5, 10, and 15 min for SNPs administration; 10 min for dissolved CBD), the animals were euthanized by CO_2_ aspiration. The brain was carefully excised, frozen at −80 °C, lyophilized overnight, and kept at −20 °C. Just before analysis, the dry brain tissue was crushed, extracted with 2 mL methanol, sonicated for 8 min, centrifuged at 17,000 rcf for 12 min, and the separated supernatant was analyzed by HPLC.

### 2.13. Statistical Analysis

Data were derived from at least two independent experiments performed in triplicate and expressed as mean values (±SD). The statistical differences between mean values were assessed by using the Student’s *t*-test at *p* < 0.05.

## 3. Results and Discussion

### 3.1. Production and Size Characterization of Nanoparticles

**Figure 1:**
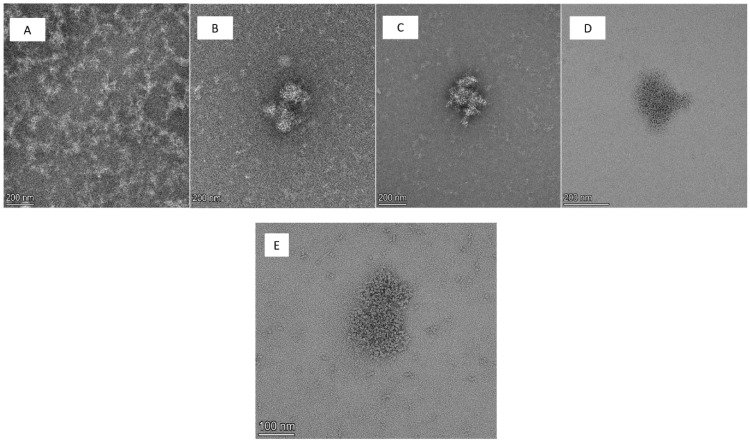
TEM images of CBD-loaded starch nanoparticles, non-crosslinked (**A**), and crosslinked with 5% DV (**B**), 10% DV (**C**), 15% DV (**D**) at ×45,000 magnification. Starch nanoparticles crosslinked with 10% DV (**E**) are also shown at ×92,000 magnification, demonstrating a cluster composed of smaller nanoparticles.

Based on a previous study in our group [15], we set out to develop a starch-based nanoparticulate system loaded with cannabidiol (CBD). As mentioned previously, CBD possesses anti-inflammatory and neuroprotective effects which are highly relevant for the treatment of neurodegenerative diseases [8,9,17]. Starch was chosen as a particle-forming polymer because it is a natural, renewable, and biodegradable substance, and is widely used in various pharmaceutical applications [14,18,19]. Following SNP production, we first characterized these nanoparticles by analyzing particle size, showing that the initial process yielded nanoparticles larger than 200 nm. Interestingly, by decreasing starch concentration in the aqueous solution before nanoprecipitation from 1.7 mg/mL to 1mg/mL, the average size was reduced (Table 1). Table 1 summarizes the size measurements of nanoparticles crosslinked by divanillin (DV) at two concentrations, demonstrating that size reduction was affected by decreasing the starch concentration only, and was not dependent on the crosslinkage degree. However, the use of 20% crosslinker on a starch basis at 1.7 mg/mL of starch concentration (in the aqueous solution prior to the nanoprecipitation) produced an average particle size of >300 nm and this formulation was not examined further. This phenomenon of reduction in particle size while the starch concentration decreased is in agreement with previous publications [20,21]. Hedayati et al. [20] suggested that the high viscosity of a highly concentrated starch gel solution hinders the dispersion of starch slurry toward the non-solvent, leading to the formation of larger particles. Saari et al. [21] attributed this phenomenon not to the particle size, but to an aggregation of smaller nanoparticles, suggesting that at higher concentrations, more particles of a similar size are formed and aggregated to produce clusters. In the present study, we have shown by using TEM imaging that the nanoparticle size measured by NTA is indeed related to a cluster of aggregated small nanoparticles (Figure 1). As shown, the crosslinking did not significantly change the size of the SNP clusters, however: as evidenced by TEM images, it formed denser cluster structures compared to the non-crosslinked SNPs.

We chose a formulation that was produced with an initial starch concentration of 1 mg/mL for all our subsequent experiments, since the mean particle size was smaller compared to the formulation with a higher initial starch concentration. Crosslinking agents can enhance the stability of the formulation. They can also influence the size of the nanoparticles that are formed [22]. Crosslinker presence can also change the nanoparticle morphology [23]. Since these factors can have an influence on tissue penetration or biodegradation, we have decided to explore both the non-crosslinked and crosslinked formulations. The uploading of CBD to the nanoparticles was monitored using an extraction technique involving a mixture of equal volumes of water and methanol. It was realized that the extraction of CBD from the dried SNP powder was more efficient using this technique than by using 1% Triton-X 100, N-methyl-2-pyrrolidone (Pharmasolve™), methanol only, or even 9:1 methanol-water. The entrapment efficiency (EE%) of CBD in the nanoparticles was determined, and the values were 39.4% ± 13.9, 29.5% ± 5.08, 29.2% ± 10.6, and 32.5% ± 8.2 for SNPs crosslinked with 0%, 5%, 10%, and 15% DV, respectively. It was also found that the EE% was higher when the nanoprecipitation process was performed at pH 3. Starch solutions with a pH lower or higher than 3 resulted in a decrease in the CBD loading (30% reduction in the crosslinked SNPs and 25% in the non-crosslinked SNPs). These data were obtained during a preliminary evaluation where one batch preparation was performed for each of four formulations (1 mg/mL initial starch; 0%, 5%, 10% and 15% DV) at 4 different pH conditions (pH = 2.3, 2.5, 3, 4.4). As a result of this experiment, the EE was consistently higher at pH 3 for all formulations. Such influence of pH on nanoprecipitation processes was also reported by Podaralla and Perumal [24]. As shown above, the EE values of the crosslinked SNPs were lower than the non-crosslinked SNPs (29.2–32.5% vs. 39.4%). This expected finding indicates that the crosslinking of starch by divanillin decreased the intermolecular space of the amorphous regions in the polysaccharidic structure.

### 3.2. CBD Penetration to the Brain after Intranasal Administration

**Figure 2:**
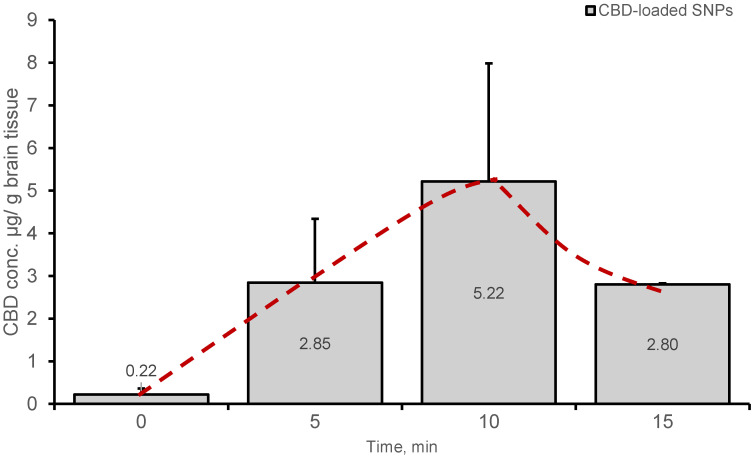
Brain levels of CBD after IN administration of CBD-loaded SNPs 5% DV—100 µg dose, 0.4 mg/kg body weight. Mean (±SD) measured in methanolic brain extracts, n = 2 for 0 and 15 min, n = 3 for 5 min, n = 6 for 10 min. Animals were randomly assigned to each group.

It was hypothesized that a nanoparticulate system is more advantageous than a solution in delivering a drug to the brain following intranasal administration (IN). Preliminary data obtained by brain CBD analyses following IN administration of SNPs and a CBD solution to rats (Section 2.12) supported this hypothesis. Figure 2 presents the results of the preliminary pharmacokinetic study of brain CBD after IN administration of CBD-loaded crosslinked SNPs (5% DV) in rats (100 µg CBD dose, n = 6). The peak brain concentration (*C*_max_ = 5.22 μg/g tissue) was determined 10 min after administration. In contrast, the concentrations of brain CBD after IN administration of non-particulate CBD, which had been dissolved in PEG 400: saline: ethanol (100 µg dose of CBD in solution, n = 3) at the same time points, were under the limit of detection of the HPLC method, namely, less than 10 nanograms/mL. Conventional formulations administered intranasally rely on absorption through the nasal epithelium into the blood circulation. This may allow us to avoid the hepatic first pass effect [25], yet the delivery into the brain is dependent on the brain blood vessels that create the blood brain barrier. As a consequence of this systemic route, plasma *t*_max_ following IN administration of CBD solution in rats and dogs was detected at approximately 30 min [16,25]. In contrast, when nanoparticulate-aided administration is employed, the major anticipated route is through the olfactory nerves directly into the brain [10]. This faster and more efficient penetration into the brain is due to the circumvention of the systemic circulation by direct trafficking into the CNS. Extensive pharmacokinetic studies, which are beyond the scope of this report, are needed to elaborate and establish the mechanism in which SNPs target the brain following IN administration. These studies will be separately published.

### 3.3. CBD Release from SNPs

The release of CBD from SNPs was monitored in a culture growth medium without dissolution excipients such as bovine serum albumin. Cumulative released concentrations of CBD were measured at various periods of time after dispersion of CBD-loaded SNPs in the medium, with and without stirring. The measurements revealed that the release of CBD from the SNPs was incomplete while the concentration of CBD released to the medium accounted for 44% and 38% of total applied CBD in stirred and unstirred samples, respectively (at a peak reached after 2 h). A total of 40% and 31% of the applied CBD in the SNPs were obtained immediately upon the application of stirred and unstirred samples, respectively. After two hours of release study, the CBD concentration in the medium slowly declined. This phenomenon may result from a two-way process, dissociation of CBD from the SNPs and its precipitation, or reattachment to the available SNPs in the media. The dissociation is initially dominant, followed by an equilibrium state. A graphical description of the experiment is found in Supplementary Materials, Figure S1.

### 3.4. Cellular Uptake of CBD-SNPs

The experimental procedure for the evaluation of nanoparticle entry into cells has previously been described by Zamansky et al. [26]. In these experiments we used BV2 cells in culture and CBD-loaded SNPs which had been crosslinked with 15% DV. Briefly, after 24 h treatment, the BV2 cells in each well were washed, detached from the wells, counted, and divided into three equal fractions treated with either PBS×1, Triton-X 100 1%, or methanol 100%. The purpose of the treatments was to retrieve CBD from separate parts of the cells: (a) PBS to recover CBD from the cell surface (“ExCell” fraction), (b) Triton X-100 to recover CBD from the cell surface and cytosol (CBD within cells, but not in the nucleus), and (c) methanol to extract total CBD. By subtracting the CBD quantity found in the ExCell fraction from the amount found by Triton X-100, the amount within the cytosol (“InCytosol”) is determined. By extracting the CBD quantity found in ExCell fraction from the total CBD quantity as analyzed in the methanol extract, the amount within the cells (“InCell”; cytosol + nucleus) is determined. Cell viability was examined using trypan blue staining. The obtained viability data were 83.5% ± 4.9, 88.5% ± 12.0, and 69% ± 1 for untreated control, 5 µM CBD and 10 µM CBD concentrations in SNP dispersion, respectively (n = 3). There was a statistically significant difference between the control cells and the cells treated with 10 µM CBD concentration in SNPs as well as between cells treated with 5 µM and 10 µM CBD concentrations in SNPs. No significant difference between the control and 5 µM treatment groups was discovered. The lower viability obtained in the 10 µM CBD-SNP treated cells as determined by trypan blue staining (18% reduction) was different from the results acquired by the MTT viability test which demonstrated viability reduction only at 15 µM CBD in SNPs, probably because the trypan blue viability values were not calculated relative to the control cells. As shown in Table 2, the quantity of CBD found in the “ExCell” fraction was, as expected, similar in all experiments, as the concentrations of nanoparticles were approximately the same. This supports previous studies showing that CBD exhibits some of its effects through interactions with the cell membrane receptors, such as the CB1, CB2, TRP receptors, GPR receptors, 5-HT_1A_ receptor, and A_2A_ receptor [8,27]. Furthermore, it may indicate that there was an interaction between CBD-loaded SNPs and the surface layer of the cells. When the low concentration of CBD in SNPs dispersion (5 μM) was applied to cells, “InCell” and “InCytosol” CBD fractions decreased significantly compared to the higher concentration of CBD in SNPs. However, no differences between “InCell” and “InCytosol” CBD fractions were found, suggesting that CBD entering the cytoplasm did not cross the nuclear membrane. CBD in the cytosol can influence NADPH oxidase, NF-kB and STAT3 pathways, or act as an agonist on the PPARƴ receptor on the nuclear membrane [27,28].

### 3.5. Effect of CBD-Loaded SNPs Treatment on Cell Viability and Nitric Oxide (NO) Formation in BV2 Cells

Concurrently with neuroprotective and anti-inflammatory effects, there are also a few reports indicating that CBD has a cytotoxic effect on cancer and normal cell lines [29,30,31]. The effect on the viability of microglial BV2 cells in normal growth conditions was evaluated for CBD-loaded SNPs and for matching numbers of unloaded SNPs. We found a significant reduction in viability at concentrations of ≥15 µM CBD in SNPs with more than a 50% reduction in viability at a concentration of 25 µM of CBD in SNPs. This effect is in accordance with a report describing a reduction in the viability of normal human cells following a treatment with CBD at concentrations greater than 10 µM [32]. Unloaded SNPs caused a certain growth inhibition (15–20%) only with a particle concentration of 4 × 10^9^ nanoparticles per milliliter, which corresponded to the concentration of particles for 25 µM CBD treatment. In contrast, the viability of LPS-inflamed cells was significantly reduced at a concentration of 10 µM CBD in SNPs, that is, 53% ± 20.7, *p* < 0.02 and 55% ± 12.8, *p* < 0.004 (mean ± SD; n = 9; three independent experiments) relative to untreated control of LPS-induced cells (‘LPS control’) for non-crosslinked (0% DV) and crosslinked (15% DV) starch formulations, respectively. This can be attributed to an increase in cytotoxic effect of CBD under conditions of a reduced serum in the growth medium as described by Sainz-Cort et al. [31]. We further conducted experiments using 5 µM and 7 µM of CBD in SNP dispersion and matching numbers of unloaded nanoparticles of the same formulations (0% and 15% DV). After treatment of LPS-induced cells with CBD-loaded SNPs we found no significant reduction in viability; however, reduced viability was noted only after treatment with non-crosslinked SNPs loaded with 7 µM CBD concentration, 73.2% ± 15.9, *p* < 0.05 (mean ± SD; n = 9; 3 independent experiments), relative to “LPS control”. This reduction, which was related to the absence of crosslinker in the formulation, may be due to a higher release of CBD to the medium and to a more prominent cytotoxic effect. A slight elevation in viability (3–5%, *p* < 0.05) was recorded for 2 out of 4 groups of unloaded SNPs, while the other groups were not statistically different from the “LPS control”. There are inconsistent data regarding the cytotoxic concentration of CBD in microglia cells under inflammatory conditions. Dos-Santos-Pereira et al. [33] mentioned a successful use of CBD at concentrations up to 10 µM in primary microglia cells for the prevention of LPS-induced inflammation, while Kozela et al. [9] showed only a slight reduction in viability when applying 10 µM CBD to LPS-inflamed BV2 cells. On the other hand, a report by Wang et al. [34] demonstrated around a 60% reduction in viability of microglia BV2 cells occurred using the same CBD concentration and same time frame of treatment as in the study of Dos-Santos-Pereira et al. [33]. Nonetheless, it is difficult to utterly compare these reports since some experimental conditions, such as the LPS concentration and the time of LPS and CBD treatment, were different. In addition, no direct comparison can be made between the current use of CBD-loaded SNPs and the CBD-containing solutions that were used in the published experiments. Detailed information regarding viability experiments is found in Supplementary Materials, Figures S2–S4.

**Figure 3:**
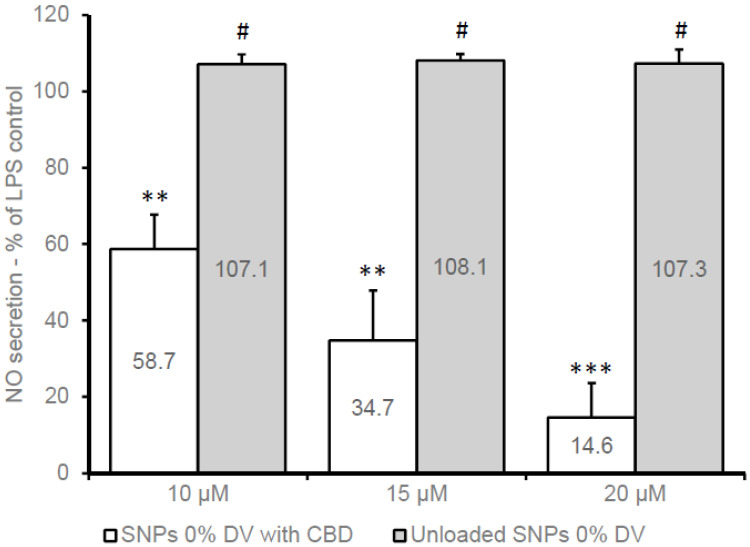
Nitric oxide secretion in BV2 cells 22 h after treatment with CBD-loaded and unloaded SNPs in medium with 1% FCS and LPS (7 ng/mL). Mean (±SD) of three independent experiments (n = 9). ** *p* < 0.005 against LPS control; *** *p* < 0.0002 against LPS control; # not significant against LPS control.

**Figure 4:**
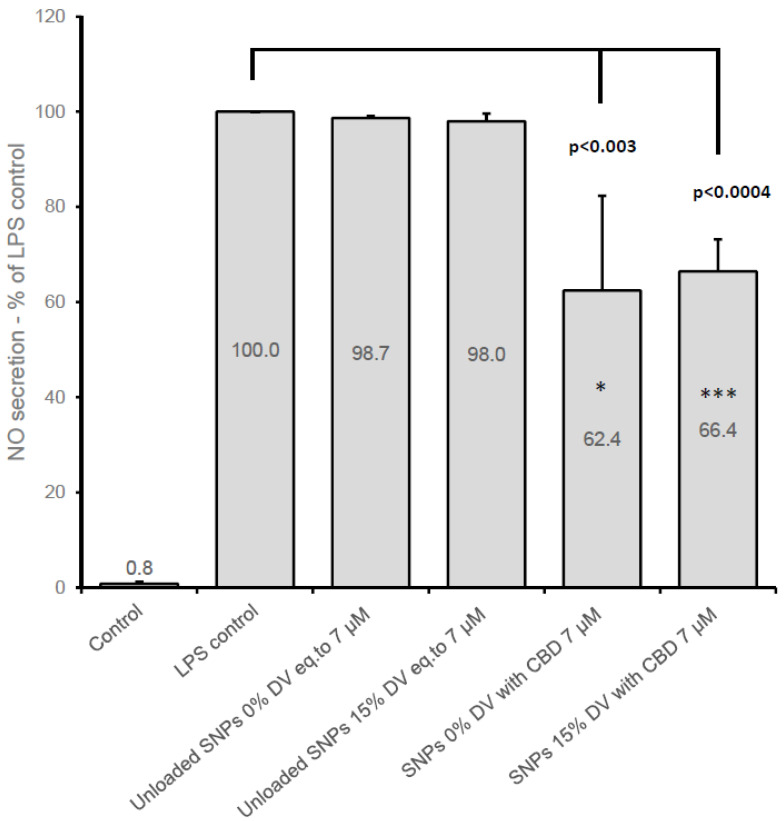
Nitric oxide secretion in BV2 cells 22 h after treatment with CBD-loaded and unloaded SNPs (two formulations)in medium with 1% FCS and LPS (7 ng/mL). Mean (±SD) of three independent experiments (n = 6 for control, LPS control, and unloaded SNPs; n = 9 for CBD-loaded SNPs). * *p* < 0.03 against matching blank SNPs; *** *p* < 0.0006 against matching blank SNPs. Values were adjusted for cell viability in case the viability was lower than 95% of LPS control group.

Under consideration based on the cell viability testing, the nitric oxide (NO) formation was evaluated in inflamed (LPS-induced) BV2 cells treated with either unloaded or CBD-loaded SNPs (with 0% or 15% DV). CBD-loaded SNPs were compared to unloaded SNPs (set to contain a matching number of nanoparticles per milliliter), in LPS-induced (7 ng/mL) cells and non-inflamed control cells (treated with a medium without LPS). SNPs unloaded with CBD did not affect NO secretion (Figure 3), whereas a concentration-dependent reduction in NO production was observed with non-crosslinked SNPs (0% DV) containing 10, 15, and 20 µM CBD, resulting in 58.7% (±9.0%), 34.7% (±13.0%), and 14.6% (±9.0%) of released NO (n = 9) relative to the NO formed by the LPS-inflamed control cells, respectively (Figure 3). However, as mentioned earlier, cell viability was compromised at these CBD concentrations, and therefore further cell inflammation experiments were conducted using lower CBD concentrations. Figure 4 presents an experiment with two SNP formulations (0% and 15% DV) containing CBD at a concentration of 7 µM. The reduction in NO production was approximately 35% for both formulations compared to NO release from untreated LPS-induced control cells (Figure 4). The use of 5 µM CBD in SNPs at the same conditions in three independent experiments has also resulted in a statistically significant reduction of NO formation (obtained values of 84.1% ± 9.3, *p* < 0.03 and 86.3% ± 5.6, *p* < 0.01 for 0% DV and 15% DV, respectively) relative to NO release from LPS-induced control cells (n = 9).

### 3.6. Effect of Treatment with CBD-Loaded SNPs on IL-6 Levels in BV2 Cells

**Figure 5:**
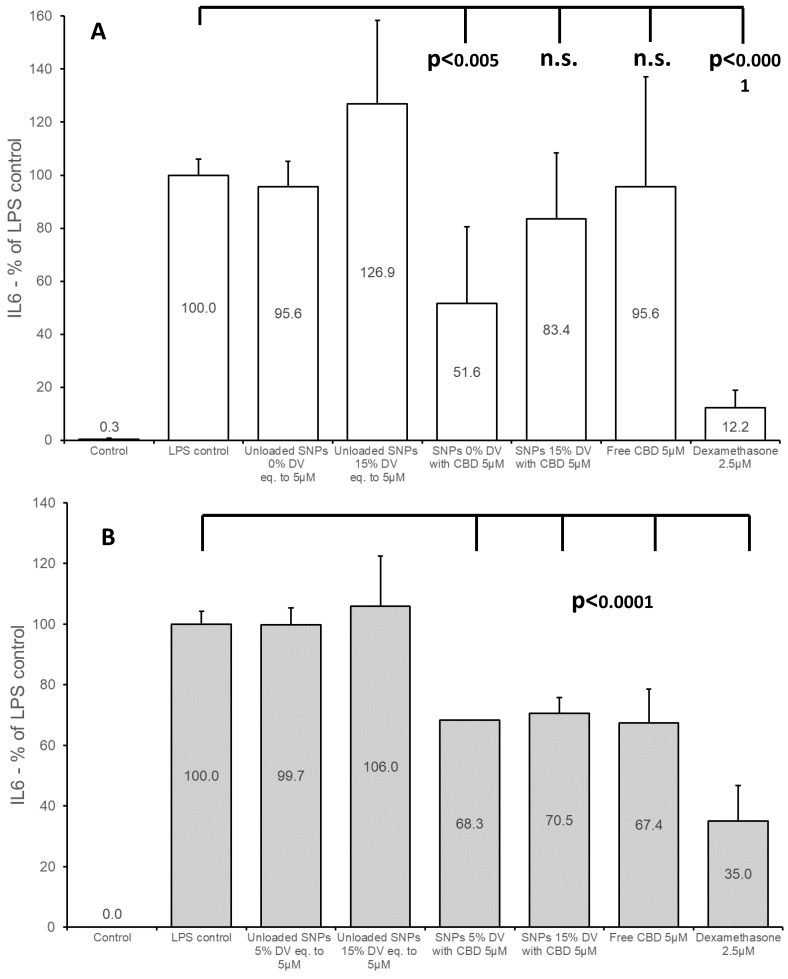
IL-6 production in BV2 cells 22 h after treatment with CBD-loaded and unloaded SNPs. The medium was prepared with 1% FCS, LPS (7 ng/mL), and 0.1% BSA (**A**) or 0.25% BSA (**B**). Mean (±SD) of two or three independent experiments (n = 6).

The secreted levels of IL-6 were also monitored. IL-6 is an interleukin produced throughout the inflammation process in the medium of BV2 cells and used as an additional marker of inflammation level. As expected, a significant elevation in the level of IL-6 was noted as compared to the untreated control 22 h after LPS stimulation (7 ng/mL) which had been preceded by a 4 h “starvation period” (see Materials and Methods section). The glucocorticoid dexamethasone (2.5 µM) was used as a positive control for anti-inflammatory effect [33]. The utilization of SNP formulations containing 5 µM CBD without crosslinker resulted in the reduction of IL-6 levels by approximately 50% as compared to LPS-treated control cells (“LPS control”) using an experimental procedure identical to that of NO experiments (Figure 5A). Although there was an apparent reduction in IL-6 when crosslinked (15% DV) SNPs were applied to the cells at the same CBD concentration (83.4 ± 25% relative to “LPS control”), this reduction was not statistically significant. However, it was noted that unloaded nanoparticles of this formulation (SNPs with 15% DV) caused an elevation in IL-6 (126.9% ± 31) compared to the “LPS control”. Two additional controls, CBD solution in DMSO diluted with experimental medium (“free CBD”; 0.18% final DMSO concentration) and dexamethasone, were also examined. Interestingly, we did not observe any effect of the “free CBD” on IL-6, although it was used at the same concentration as in the SNP formulations. CBD is practically insoluble in water but it is solubilized in BSA-supplemented growth medium (up to 8–10 µM), and the solubility can be further increased with additional BSA supplementation [35]. The experimental medium used in the present study contained 0.1% *w*/*v* BSA. We assumed that the reason for the lack of effect of 15% DV formulation as well as the “free CBD” was a relatively lower availability of CBD to the cells, either by immediate precipitation of the “free CBD” or by the slow rate of release of CBD from the crosslinked SNPs. As was demonstrated by Zamansky et al. [35], the addition of BSA can increase the release of CBD from nanoparticles and improve its solubility. We further confirmed that the CBD release from SNPs dispersed in water supplemented with BSA was improved. CBD release in purified water with 0.25% *w*/*v* BSA was increased by 64%, 90%, and 111% as compared to 0.1% *w*/*v* BSA at 0, 60, and 120 min, respectively. Therefore, we conducted a further series of experiments with 0.25% *w*/*v* BSA in the experimental medium (Section 2.9 and Section 2.10). This addition resulted in a significant reduction of IL-6 after the “free CBD” treatment, but not after treatment with the crosslinked SNPs. We hypothesized that the possible cause of this phenomenon is related to the increase in IL-6 caused by unloaded crosslinked SNPs. Treatment with unloaded SNPs at a concentration of 1.34 × 10^9^ nanoparticles per milliliter (which was approximately equal to the concentration of nanoparticles of a similar formulation loaded with 5 µM CBD) resulted in IL-6 values of 138% ± 34 of the untreated control (LPS-induced cells). This finding is in accordance with the result mentioned earlier (126%). However, treatment of LPS-induced cells with a crosslinked starch formulation in which the nanoparticle concentration was reduced by half resulted in a reduction of IL-6 to levels compared to the LPS-treated control. It should be noted that the treatment of non-LPS-induced cells with a crosslinked starch formulation, even at an increased nanoparticle concentration (×2), did not cause IL-6 elevation. We, therefore, produced SNPs (15% DV) using an increased *in-process* CBD quantity (×2.5). Although the EE of the formulation decreased to 23%, this resulted in a higher CBD concentration (in µg CBD/mg SNPs) and enabled treatment with the same concentration of CBD as in the previous assays, but with a reduced nanoparticle concentration (in units/mL). Figure 5B summarizes these experiments. As shown, the use of 15% DV in CBD-loaded SNP formulation resulted in a reduction of IL-6 by approximately 30%, compared to the untreated control of LPS-induced cells (“LPS control”). A similar reduction was noted in the “free CBD” group. The use of 5% DV containing formulation exhibited comparable results to 15% DV containing formulation and “free CBD”. There was no significant difference between “LPS control” and the unloaded SNP formulations (*p* = 0.94 and *p* = 0.34 for 5% and 15% DV-containing formulations, respectively). Viability was not compromised in any of the groups represented in Figure 5.

**Figure 6:**
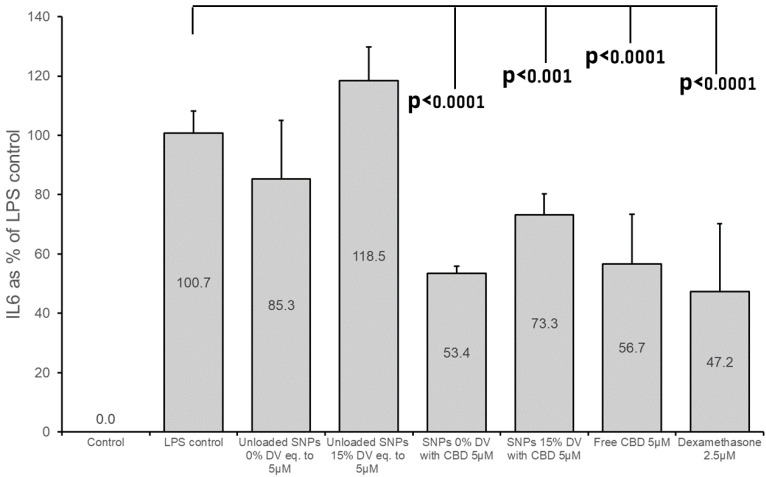
IL-6 production in BV2 cells 6 h after treatment with CBD-loaded and unloaded SNPs. The medium was prepared with 1% FCS, LPS (7 ng/mL) and 0.25% BSA. Mean (±SD) of two or three independent experiments (n = 6 for control, unloaded SNPs, CBD-loaded SNPs, and dexamethasone; n = 8 for LPS control and free CBD).

It was previously demonstrated that IL-6 levels in BV2 cells started to rise approximately 2 h after LPS stimulation, continued to rise and remained at high levels 24 h after stimulation [36]. In the present study, we examined both the post-treatment effect of SNPs at 22 h after LPS stimulation (Figure 5), and 6 h after the stimulation (Figure 6). As evident from Figure 6, the anti-inflammatory effect is also noted at 6 h after treatment. A decrease of approximately 45% in IL-6 formation was observed after treatment with CBD-loaded non-crosslinked SNPs, which was comparable to the effect of “free CBD”. A reduction of more than 25% was noted for CBD-loaded crosslinked SNPs (15% DV). The 15% reduction in IL-6 after treatment with unloaded non-crosslinked SNPs was found not to be statistically significant (*p* = 0.09); however, a significant increase (*p* = 0.02) in IL-6 formation was measured after treatment with unloaded crosslinked SNPs at this time point. This 18% increase may explicate the relatively lower reduction in IL-6 level after treatment with the CBD-loaded crosslinked (15% DV) SNPs in this experiment.

Muñoz et al. [37] reported that size-dependent inflammation was initiated by nanoparticles due to the interaction with cell membranes. The inflammation was attributed to smaller-sized (10–40 nm) particles while larger particles (100–1000 nm) behaved rather inertly [37]. As reported in the present study (Table 1 and Figure 1), all SNP types were structured in clusters of a similar size; however, the crosslinked SNPs created a denser cluster of nanoparticles compared to the loosely connected clusters created by the non-crosslinked SNPs. The denser structures of the crosslinked SNPs may imply a general tendency of these nanoparticles to pursue interfacial reactions with interactive surfaces such as cell membranes, which resulted in increased IL-6 release.

## 4. Conclusions

Cannabinoids, including cannabidiol (CBD), are potential active agents for the treatment of inflammation-related conditions, with an emphasis on neuroinflammation. In this study, starch-based CBD-containing nanoparticles were produced with and without the addition of a crosslinker. The adjustments of several parameters during production resulted in improved particle characteristics in the size and entrapment of CBD. A preliminary in vivo study indicates an advantage of intranasal CBD-loaded SNPs over intranasal CBD in solution regarding brain penetration. Additional examination has demonstrated the ability of these SNPs to deliver entrapped CBD into microglia cells. This action resulted in a reduction of NO production and IL-6 levels indicating an anti-inflammatory activity without a decrease in viability.

## Figures and Tables

**Table 1:** Summary of the mean particle size of CBD-loaded starch nanoparticles manufactured at two starch concentrations and several crosslinking degrees. (* statistically significant, *p* < 0.05; ^NS^ not statistically significant).

Formulation	Aqueous Starch Concentration, mg/mL	Divanillin Concentration on Starch Basis, %*w*/*w*	Mean Particle Size ± SD (in Diameter), nm	Polydispersity Index
SNP0-1	1.7	0	274.0 ± 75.2	0.11 ± 0.03
SNP0-2	1.0	0	* 187.4 ± 19.3	0.19 ± 0.03
SNP5-1	1.7	5.0	260.5 ± 16.1	0.15 ± 0.07
SNP5-2	1.0	5.0	* 206.1 ± 21.0	0.13 ± 0.02
SNP10-1	1.7	10.0	248.6 ± 11.8	0.16 ± 0.07
SNP10-2	1.0	10.0	^NS^ 205.8 ± 44.4	0.17 ± 0.06
SNP15-1	1.7	15.0	297.0 ± 14.8	0.16 ± 0.02
SNP15-2	1.0	15.0	* 206.7 ± 43.6	0.21 ± 0.07

**Table 2:** Cellular uptake of CBD from CBD-loaded SNPs (15% DV) in BV2 cell culture, 24 h after treatment (values presented as mean ± SD).

	10 µM CBD 2.67 × 10^9^ SNPs/mL	5 µM CBD 1.33 × 10^9^ SNPs/mL
“ExCell” CBD, µg	0.15 (±0.002)	0.15 (±0.008)
“InCell” CBD, µg	0.21 (±0.05)	0.12 (±0.02)
“InCytosol” CBD, µg	0.22 (±0.04)	0.12 (±0.04)
% of administered CBD found in cells	1.4	1.6

## Data Availability

Data can be available under request.

## Supplementary Materials

The following supporting information can be downloaded at: https://www.mdpi.com/article/10.3390/pharmaceutics15071803/s1, Figure S1: Time dependent CBD release from SNPs in growth medium. One sample at each time point was analyzed, without and with stirring. CBD-loaded SNPs 15% DV. Expected CBD concentration in case of total release of SNPs-loaded CBD–2.3 µg/ml; Figure S2: BV2 cells viability 22 h after treatment with CBD-loaded SNPs (two formulations and various CBD concentrations) in normal growth conditions. Mean (±SD) of three independent experiments (n = 30 for control (not presented); n = 15 for each CBD-loaded SNPs group). *—*p* < 0.01 against untreated control; **—*p* < 0.002 against untreated control; ***—*p* < 0.0009 against untreated control; Figure S3: BV2 cells viability 22 h after treatment with CBD-loaded and unloaded SNPs (two formulations)—medium with 1% FCS and LPS (7 ng/mL). Mean (SD) of three independent experiments (n = 6 for control, LPS control (not presented) and unloaded SNPs; n = 9 for CBD-loaded SNPs). *—*p* < 0.05 against LPS control; #—nonsignificant against LPS control; Figure S4: BV2 cells viability 22 h after treatment with CBD-loaded and unloaded (blank) SNPs (two formulations)—medium with 1% FCS and LPS (7 ng/mL). Mean (±SD) of three independent experiments (n = 18 for control and LPS control; n = 9 for each treatment group). *—*p* < 0.02 against LPS control; **—*p* < 0.006 against LPS control; ***—*p* < 0.0009 against LPS control; #—nonsignificant against LPS control.
