# Supplementary material for: Cannabidiol-Loaded Nanoparticles Based on Crosslinked Starch: Anti-Inflammatory Activity and Improved Nose-to-Brain Delivery
Source: Pharmaceutics. 2023 Jun 23;15(7):1803. doi: 10.3390/pharmaceutics15071803 (PMC10384644; doi:10.3390/pharmaceutics15071803)
Supplement: Supplementary file 1 [file pharmaceutics-15-01803-s001.zip › pharmaceutics-2441429-supplementary.pdf]

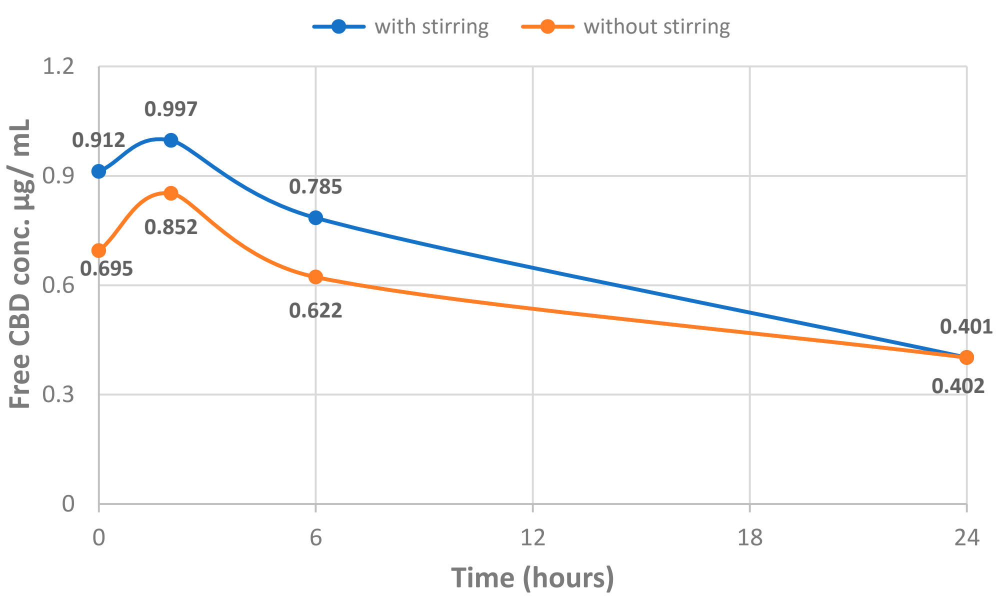

Figure S1: Time dependent CBD release from SNPs in growth medium. One sample at each time point was analyzed, without and with stirring. CBD-loaded SNPs 15% DV. Expected CBD concentration in case of total release of SNPs-loaded CBD–2.3 µg/ml.

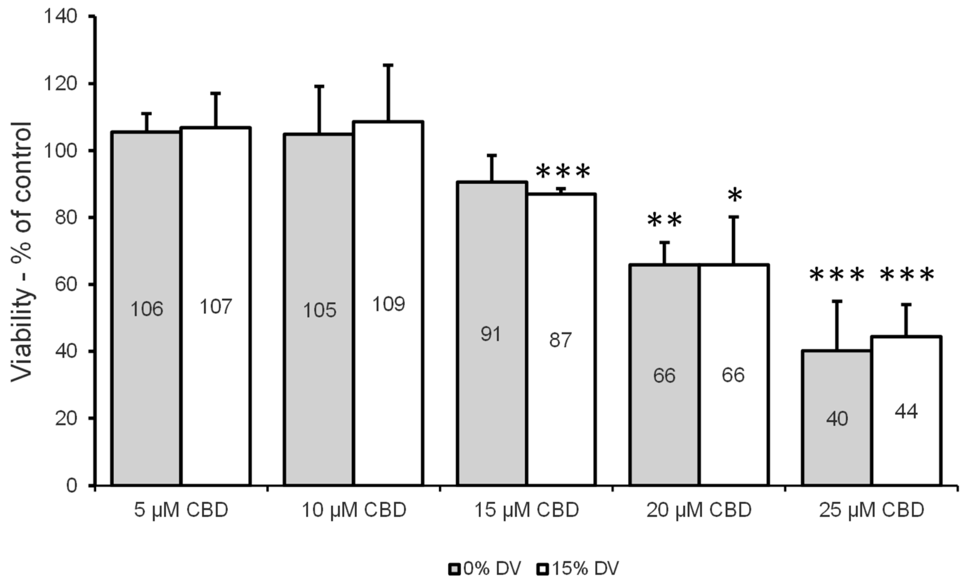

Figure S2: BV2 cells viability 22 h after treatment with CBD-loaded SNPs (two formulations and various CBD concentrations) in normal growth conditions. Mean (±SD) of three independent experiments (n = 30 for control (not presented); n = 15 for each CBD-loaded SNPs group).

*—$p < 0.01$ against untreated control; **—$p < 0.002$ against untreated control; ***—$p < 0.0009$ against untreated control.

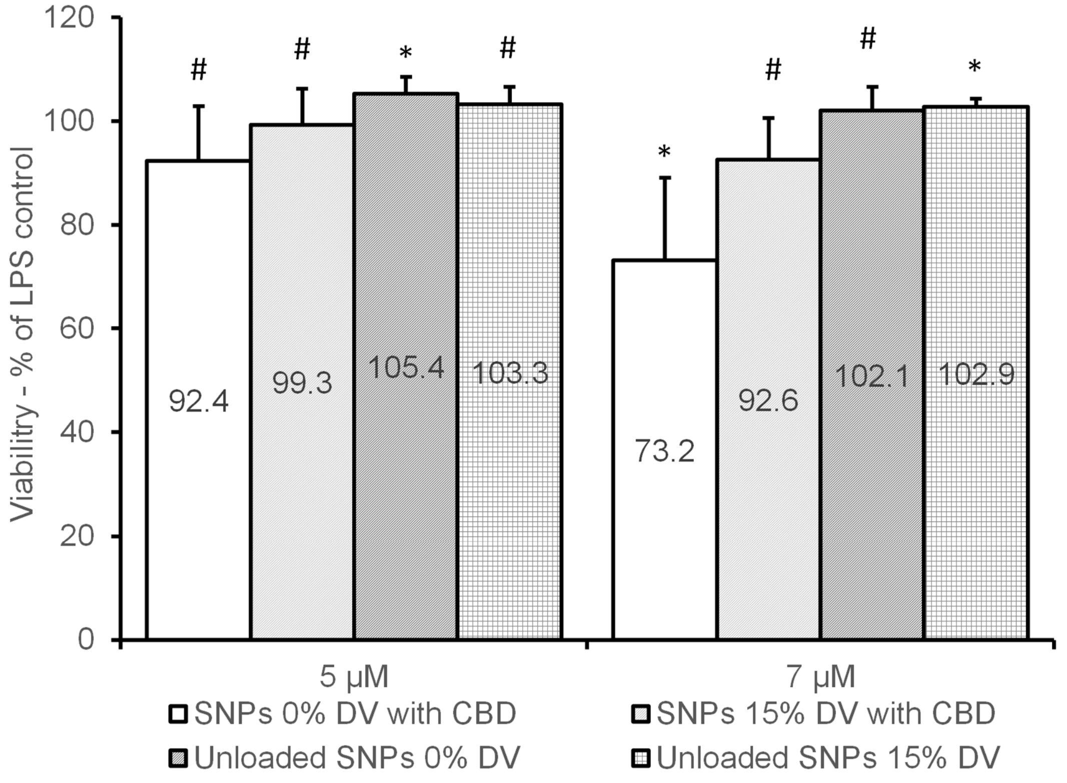

Figure S3: BV2 cells viability 22 h after treatment with CBD-loaded and unloaded SNPs (two formulations)—medium with 1% FCS and LPS (7 ng/ mL). Mean (SD) of three independent experiments (n = 6 for control, LPS control (not presented) and unloaded SNPs; n = 9 for CBD-loaded SNPs). *—$p < 0.05$ against LPS control; #—nonsignificant against LPS control.

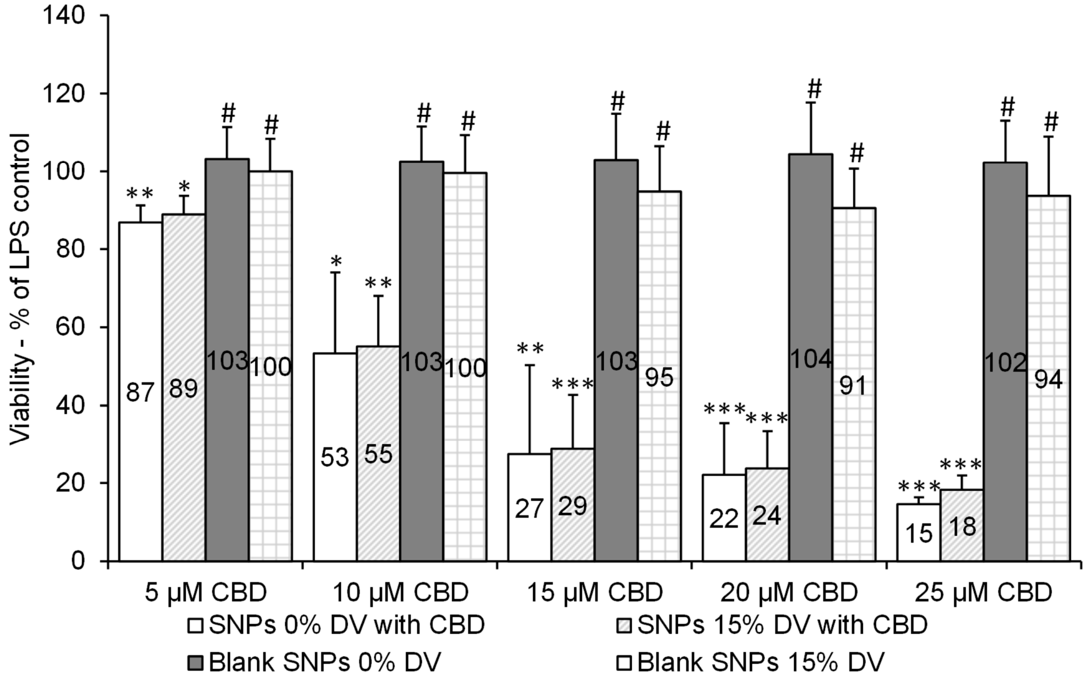

Figure S4: BV2 cells viability 22 h after treatment with CBD-loaded and unloaded (blank) SNPs (two formulations)—medium with 1% FCS and LPS (7 ng/ mL). Mean (±SD) of three independent experiments (n = 18 for control and LPS control; n = 9 for each treatment group). *—$p < 0.02$ against LPS control; **—$p < 0.006$ against LPS control; ***—$p < 0.0009$ against LPS control; #—nonsignificant against LPS control.
